# Socioeconomic Profile of Tourists with a Greater Circular Attitude and Behaviour in Hotels of a Sun and Beach Destination

**DOI:** 10.3390/ijerph17249392

**Published:** 2020-12-15

**Authors:** Carlos Rodríguez, Marta Jacob, Carmen Florido

**Affiliations:** 1 Department of Applied Economic Analysis, University of Las Palmas de Gran Canaria, 35017 Las Palmas de Gran Canaria, Spain; carmen.florido@ulpgc.es; 2 Department of Applied Economics, University of the Balearic Islands, E-07122 Palma, Spain; marta.jacob@uib.es

**Keywords:** circular economy, tourist behaviour, tourist attitude, hotel industry, sustainability, destination

## Abstract

This work aims to analyse the attitude towards circular economy (CE) and the environmental behaviour and circular practices among tourists of a well-known mature sun and beach destination. The study was conducted on a sample of tourists who visited Gran Canaria and stayed at a hotel establishment. Findings show that: (a) Older tourists have a higher pro-environmental or circular attitude in hotel establishments than younger ones; (b) most tourists are willing to pay more for environmentally friendly or green hotels and this is related to socio-demographic variables; (c) the majority of tourists believe that it is important for the hotel to have an energy-saving policy; (d) tourists’ attitude towards circular practices varies according to socio-economic profile; (e) the most common sustainable hotel practices carried out by tourists are the use of recycling bins and reusable towel and linen schemes; (f) women report a higher circular behaviour than men; and (g) 86.5% of tourists carry out the same CE practices on holidays as in their place of residence. Study findings could be useful to design the transition from a linear model to a circular model in the hotel industry of a destination as it identifies the areas that the industry must promote to reach this transition.

## 1. Introduction

Since the end of the 20th century, different approaches related to sustainability have been developed, such as sustainable development, ecological growth, blue economy and green economy, but all of them are built upon a linear production–consumption model, based on the growth and scarcity of resources together with climate change and environmental degradation.

The circular economy (CE) agrees with these approaches focusing on the relationship of human beings with their environment but differs in that it is more radical. In fact, it requires a much wider and complete design of solutions throughout the life cycle of the product as it is based on the creation of value through the Restoration, Regeneration and Reuse of resources [1]. The aim is to implement a new economy, based on circular production systems, generating new business models and forms of consumption that move away from property and that imply the existence of active users and passive nonconsumers. Hence, a radical change in the actual linear production models is needed to implement CE, and it also entails a radical change in the way companies, citizens and legislators behave.

However, the CE literature mainly focuses on the manufacturing sector with limited references to tourism despite the tourism sector being characterised by huge energy and water consumptions, organic and plastic waste, traffic congestion and carbon dioxide emissions and air pollution.

Coastal regions and especially island destinations such as the Canary Islands rely on the coastline for the development of their economy and tourism industry, and the life quality of these regions [2]. Tourism represents 35.2% of GDP, 40.2% of employment and accounts for 35.3% of tax collection in the Canary Islands [3]; however, tourism growth and development in the Canaries have altered the state of the coastal environment and have generated negative externalities on the environment such as seawater degradation, deterioration of fauna and flora, CO_2_ emissions and pollution, erosion and destruction of ecosystems or the depletion of natural resources, and have generated an excessive coastal urbanisation with associated problems such as the visual impact [4]. In fact, the Canary Islands lead together with the Balearic Islands, another well-known mass tourism destination, the ranking of autonomous communities with the highest waste per capita generation indicator. Hence, the Canary Islands, due to its archipelago status, is a very vulnerable destination with very limited resources. One of the most serious problems associated with the development of tourist activity in the Canary Islands is, on the one hand, the consumption of high levels of resources and, on the other, the generation of waste.

The implementation of CE models and solutions is especially important at island destinations such as the Canary Islands where an adequate and sustainable management of resources seems to be a key element in current and future tourism policies for this destination. Tourism businesses and destinations can take advantage of many CE initiatives to reduce the consumption of natural resources, organic and plastic waste generation and CO_2_ emissions; and reuse, recycle and recover products, services, waste, materials, water and energy, but also to achieve greater profitability and increased revenues in services provision, for example, in the hotel sector. 

In order to implement a transition towards a CE strategy at any destination, one must consider all the relevant actors: DMOs and key stakeholders, resident population, tourism businesses and tourists [5]. In fact, tourists’ attitude towards CE and their behaviour in terms of green, sustainable and circular practices during their holidays are crucial for a transition to a circular economy model in the tourism sector and destination. Although the hotel establishments and the destination implement actions aimed at a change in a CE model, without an adequate circular behaviour and attitude on the part of tourists, the efforts made by hoteliers and administrations will not be entirely useful. Hence, the role of consumers is a significant factor in the transition and their behaviour needs to be seen as an important contributor to the solutions [6,7,8]. Sorensen and Baerenholdt [9] indicate that tourists are co-producers of tourism experiences, and hence, the tourists’ practices sustain the transition to a circular economy. According to [10], awareness rising among tourists is essential, because that is the weakest point in the value chain. It is essential to promote a conscious attitude of tourists about the consequences of their consumption style at destinations [11]. In this sense, environmental education of tourists is important to make them aware of minimising their environmental impacts and the importance of not littering [11]. Tourists should receive more information on CE mechanisms through various marketing channels [12]. The behaviour of tourists is also essential to avoid damaging the environmental commitment assumed by tourist accommodation; therefore, collaboration between tourists and staff is needed [13].

One main research question arises regarding CE practices carried out by tourists: What socioeconomic variables affect the circular or environmentally sustainable attitude and behaviour of tourists at a mature destination? In addition, complementary research questions are raised, mainly the following: What are the most common circular practices that they carry out during their stay at the hotel? What are the most common circular practices that they carry out during their stay at a destination? This article analyses the data collected from a structured questionnaire to tourists visiting Gran Canaria (Canary Islands) and staying in hotel establishments.

The aim of this paper is to study the attitude towards CE and the environmental behaviour and circular practices among tourists of a well-known mature sun and beach destination, Gran Canaria, with serious sustainability problems, especially in coastal municipalities. Hence, to identify the socioeconomic profile of tourists with a greater circular attitude and behaviour in Gran Canaria, we will analyse different aspects such as tourists’ awareness and information and their interest or reluctance to change their practices while staying at the hotel; and the most common circular practices and those that the hotel industry must promote to reach this transition. The results could be useful to design how to move away from a linear model towards a circular model in the hotel industry of Gran Canaria and of the destination.

After the introduction, this paper is organised as follows: Section 2 describes the CE background framework, and the main literature on tourists’ attitude and behaviour towards sustainability and on circular practices carried out by tourists in hotel establishments. Section 3 outlines the main hypotheses to be tested throughout the manuscript and the underlying and supporting literature. Section 4 describes the research methodology. Section 5 shows the findings of the research; Section 5.1 shows the validity and reliability analysis; Section 5.2 describes tourist’s profile; Section 5.3 presents tourist’s travel characteristics and Section 5.4 provides information about circular practices during the tourist’s stay at the hotel and on the island and circular practices carried out at the tourist’s place of residence by testing the hypotheses outlined in Section 3. Section 6 discusses the main findings. Finally, the article concludes with a summary of future research fields and final remarks on the paper’s contribution.

## 2. Theoretical Framework

### 2.1. Circular Economy

The CE concept aims to transform the current society to a sustainable one by moving away from the actual linear ‘take, make and dispose’ economy to a closed-loop economy where there is zero waste [14].

The Ellen MacArthur Foundation has been a pioneer in popularising the concept of CE, defining it ‘as an industrial system that is restorative or regenerative by intention and design. It replaces the “end-of-life” concept with restoration, shifts towards the use of renewable energy, eliminates the use of toxic chemicals, which impair reuse, and aims for the elimination of waste through the superior design of materials, products, systems, and, within this, business models’ [1].

CE has received increasing attention between policymakers and management staff over the last decade, being a policy priority in many countries worldwide. In order to achieve the resource efficiency agenda, set by the 2030 Agenda for Sustainable Development, the European Union (EU) is on its way of transitioning from the linear economy to a circular economy model. The use, implementation and regulation of the CE have been intensified in the EU in the past years, and a specific action for resource efficiency has been designed with the ‘Resource Efficiency Roadmap’ and also the ‘Circular Economy Action Plan’ [15].

Given Europe’s concern about resource scarcity and excessive waste generation, in March 2020, the New Circular Economy Action Plan [16], for a cleaner and more competitive Europe, was published. In this Plan, the European Commission designs ‘a future-oriented agenda for achieving a cleaner and more competitive Europe in co-creation with economic actors, consumers, citizens and civil society organisations’ [17]. The goal of this Plan is to accelerate the transition required by the European Green Deal, while developing at the same time CE actions that have been implemented since 2015. This plan will ensure that the CE collaborates and cooperates with European regions and cities, as well as their citizens, contributes to reach climate neutrality and makes use of all the potential of research, innovation and this era of digitalisation.

The Circular Economy Action Plan also defines five priority areas (plastics, food waste, critical raw materials, construction and demolition, and biomass and bio-based products), which face specific challenges.

Waste generation and management are a crucial issue for a more sustainable economy. In fact, the Circular Economy Action Plan determines long-term objectives to reduce landfills and to increase the re-use and recycling of waste flows. Here, the tourism sector plays a very important role due to the greater volume of waste generated by tourists compared to the resident population. According to the European Environment Agency, tourism accounts for 6.8% of the waste generated in Europe [18]. International tourists in Europe generate around 2.8 million tons of municipal solid waste [19]. In fact, a tourist generates twice as much garbage as a resident [20]. In the Canaries, this percentage is even higher; in fact, considering a resident population of 2,106,624 persons and 14,981,113 tourists arriving to the archipelago with an average stay of 9.36 days, tourists account for 26.7% of the total waste generated on the islands [21].

The Circular Economy Action Plan of the EU is a concrete and ambitious response to the challenges for the sustainable development and for the fight against climate change. It defines a clear strategy, as well as the actions to follow to contribute to the Aims of Sustainable Development and to the Agreement of Paris on Climate change. The transition towards a CE is an advantage for the EU, as it will foster competitiveness and sustainability, building an economic system more resilient and adaptable to the shortage of material and energy resources, and to the financial volatility, promoting the innovation and managerial efficiency, and changing in a radical way the production and consumption patterns.

Regarding the tourism sector, it has a high environmental impact and can create great pressure on local resources, especially at mature tourist destinations [22]. To a large extent, all this is due to the economic model on which it is based: The linear model that prevails in today’s economy based on access to large amounts of cheap and easily accessible resources (linear model focused on extract-make-use-throw). However, despite the fact that the literature recognises that there is a scarcity and limitation of resources and that this model is no longer viable, in general, tourist destinations do not show a proactive and action-oriented policy or strategy for a transition towards a more circular tourism model [23].

Thus, to carry out the transition from a linear economy model to a circular model in the tourism sector, what is crucial, among other elements, is the awareness and knowledge of tourists, residents and enterprises about what CE implies, as without them, change is impossible. A change in the behaviour of all the actors involved is necessary. Hence, the tourists’ attitude and behaviour towards this new scenario are also important.

### 2.2. Tourists’ Attitude and Behaviour towards Circular and/or Environmentally Sustainable Practices in Hotel Establishments

Environmental sustainability is a global objective for economic activity in general and for the tourism sector in particular. Tourism contributes significantly to environmental degradation and greenhouse gas emissions [24]. The key to minimise environmental problems depends mainly on the number of people who intend to change their consumption behaviours and participate in more sustainable or circular practices with the environment. In fact, tourists’ attitude and behaviour when on holidays at a destination can help to reduce those negative impacts by making environmental decisions and behaving sustainably while staying at the destination [25]. Thus, tourists may play a central role for developing circular economy principles in tourism and specifically in hotel establishments [26,27].

Moving towards more sustainable development forms and implementing sustainable practices to guarantee long-term sustainability are a priority at any destination but especially at island destinations, where it is crucial to consider the roles of all actors involved in the destination’s management and development [5]. Thus, as Sørensen and Bærenholdt [9] stated, tourists’ images and subsequent demands for sustainable holidays and practices have to be supported by public and private actions that combine sustainable images and knowledge of CE practices in innovative solutions. These authors indicate that CE tourist practices can consist of different services and experiences and interactions with specific products/services, such as reducing water consumption while in a hotel, energy or fuel-efficient driving and food-waste recycling or minimisation. Other initiatives can also include tourists thinking in value circles, for example, by reusing, sharing, reselling and recycling material products [9].

Numerous studies indicate that tourists show interest towards the environment as a key factor in their holiday experience quality [28,29,30,31,32]. Hedlund [33], based on data from a web-based questionnaire to Swedish respondents who travelled during June–August 2007, showed a significant relationship that universalism affects environmental concerns and that environmental concerns affect the intention to buy ecologically sustainable tourism alternatives. Similarly, Kim and Choi [34] showed a positive relationship between environmentally sustainable attitude and green buying intentions. The results of Juvan and Dolnicar [25] showed that the majority of tourists who choose green transportation options choose environmental certification tourism providers or refuse to participate in tourist activities that can harm the environment.

The results of Leonidou et al. [35] show that tourist pro-environmental attitudes are key in shaping an eco-friendly behaviour; particularly, these green tourists are normally highly deontological, law-obedient and politically active. In fact, their results show that these attributes developing positive attitudes towards the environment are especially important in women, older, highly educated and of high-income level. Additionally, they indicate that tourists from Western European countries are the most environmentally friendly. Han et al. [36] found that tourists’ behaviours related to green purchasing, recycling and resource conservation depend mainly on their ethical obligation to carry out these pro-environmental actions. In fact, Kvasova [37] confirmed that personality plays a key role in shaping tourist eco-friendly behaviour.

Andereck [38] studied tourists’ perceptions of environmentally responsible practices carried out by tourism companies by analysing the attitudes of these tourists towards ‘green’ innovations in tourist sites, and he concluded that nature-oriented tourists had more positive views of environmentally responsible practices at tourist places and businesses than other tourists. Additionally, more than half of respondents believe that seeing environmentally responsible initiatives, such as recycling bins or products made from recycled materials, were really important.

On the other hand, towel and linen reuse in the hotel industry has been extensively investigated. Han and Hyun [39] analysed the behaviour of guests in relation to this field, showing that the guests’ intention to reuse towels is influenced by moral norms, social norms, anticipated feelings and the behaviour of reusing towels in everyday life. Robinot and Giannelloni [40] found that customers considered the reuse of linens and towels as a basic attribute of hotels, and not carrying out this action was considered to have a negative effect on guest satisfaction.

Some researchers have analysed visitors’ willingness to pay for conservation and protection [41,42] or willingness to pay for eco-friendly accommodations [43]. Berezan et al. [44] found that tourists normally prefer incentives for participating in a hotel’s green practices rather than paying a premium for a green-friendly hotel. Additionally, available literature illustrates that guests’ willingness to pay for environmentally friendly or green hotels is related to their socio-demographic profile, especially with age, sex and education [45,46].

Previous studies displayed that demographic differences (such as age, gender, education, nationality and income) play an important role in explaining tourists’ attitude and behaviour towards environmentally sustainable and/or circular practices in hotel establishments. Many studies show that older tourists have a more circular attitude and behaviour than younger ones [35,47,48]. Numerous studies have also demonstrated that women present higher pro-environmental behaviours than men [45,49,50,51]. Finally, available literature indicates that environmentally friendly hotel practices are correlated with nationality [30,35,44,52,53] and education [35,44,47,54,55].

There has been an environmental increase in awareness among consumers, in order to carry out the demand for actions to preserve the environment [56]. The hospitality industry is currently undergoing persistent demands from tourists to introduce environmentally friendly practices [27]. Several studies assume that those tourists with pro-environmental values and beliefs have a higher probability to behave environmentally sustainably when on vacation [57].

## 3. Hypotheses

The survey design for the research has been selected in a hypothesis-driven manner based on previous results that have emerged from the tourism and social science literature about circular or pro-environmentally sustainable tourist attitude and behaviour. The literature examined indicates that the socio-economic profile of customers may determine their attitude and behaviour regarding sustainability and circular economy issues (i.e., Barr [58]). Given this empirical evidence, the hypotheses raised for the data analysis are the following:

**Hypothesis 1** **(H1).** *Older tourists have a more circular attitude than younger tourists.*

Many studies show that older tourists have a more circular attitude than younger ones: Ayazlar and Gamze [47] found that older participants reported a more positive attitude towards green hotels than younger ones, Dolnicar [48] showed that being older is one of the best predictors of pro-environmental behaviour of tourists, and Leonidou et al. [35] found that tourists older in age are normally more eco-friendly than younger ones. As Han et al. [59] stated, several studies showed that green consumers are older tourists [60,61,62].

However, Andereck [38] found a negative correlation between age and perceived value of green practices, indicating that younger tourists place more value on environmental practices. Similarly, Kiatkawsin and Han [63] found that young people present a higher level of positive environmental attitude while Holmes et al. [64] showed that sustainable tourists are typically younger.

**Hypothesis 2** **(H2).** *Tourists are willing to pay more for environmentally friendly or green hotels. The older the tourist is, the greater the willingness to pay more.*

Berezan et al., Masau and Prideaux, and Kelly et al. [44,65,66] found positive evidence for consumers being willing to pay a premium for green hotel practices. In addition, Masau and Prideaux, and Han et al. [59,65] indicated that tourists were willing to pay more for environmentally friendly accommodations. Additionally, according to Bohdanowicz [67], almost 25% of guests in Scandinavian hotels are willing to pay more for accommodation in an eco-certified facility. Similarly, Kang et al. [68] found that those U.S. hotel guests that have a higher degree of environmental concern and awareness are more willing to pay premiums for green initiatives in hotels. A related result by Borden et al. [69] indicates that the majority of guests showed a willingness to exchange something for behaving more environmentally friendly in terms of water consumption.

On the other hand, available literature suggests that tourists’ willingness to pay for environmentally friendly or green hotels is related to their socio-demographic variables such as age, sex and education. Laroche et al. [45] proved that women were more environmentally conscious than men and were willing to pay more for green products. However, Kostakis and Sardianou [70] found that men are more likely than women to be willing to pay extra money for green hotels.

Mensah and Mensah [46] found in a sample of tourists in Ghana that most of them (83%) were willing to pay more to stay in an environmentally responsible hotel. They pointed out that only age was significantly correlated to the willingness to pay more but there were strong relationships between willingness to pay and level of education. Among people under 20 years old, 21.8% were not willing to pay more while only 9.4% were willing to pay more. However, among older people (50 years old and above), a greater percentage (10.1%) were willing to pay more compared to those who were not willing to pay (1.8%).

Other studies found different results, for example, Wehrli et al. [71] showed that respondents were not willing to pay a substantial premium for the inclusion of specific attributes. Similarly, Pulido-Fernández and Lopez-Sánchez [6] indicated that sustainable tourists might not always be willing to pay more, as they found in their study of tourists visiting Spain, and Alemão [27], Jauhari and Manaktola [72], Lee et al. [73], Baker et al. [74] and Dimara et al. [75] found that the majority of consumers were not willing to pay a premium for green practices.

**Hypothesis 3** **(H3).** *There is a positive relationship between hotel category and tourists’ awareness regarding circular economy practices.*

Larger and high-category hotels are at the frontline of environmental management in the hotel industry [76]. In fact, according to Kang et al. [68], luxury and mid-priced hotel guests are more willing to pay premiums for hotels’ green practices than economy hotel guests, so these high-category hotels can obtain higher returns from their environmental investments than low-category hotels.

**Hypothesis 4** **(H4).** *Most tourists believe that it is important for the hotel to have an energy-saving policy.*

The transition towards a CE model in the tourism sector would not be possible without the implementation of renewable energies. Ma et al. [77] argues that using renewable resources is an important element in the design of tourism circular economy. Renewable energies can be widely used in all tourism sectors (accommodation, sewage and rubbish stations, transportation, leisure and recreation, etc.) and tourism enterprises, as well as in different tourist areas and destinations.

According to Dalton et al. [78], 86% of a sample of tourists in Australian hotels support the use of renewable energy in the hotel. Additionally, survey respondents to the Deloitte Consumer Survey [79] identified the following green initiatives as the most important ones: Energy-efficient lighting (74%) and energy-efficient windows (59%), while in a study by Millar and Baloglu [80], occupancy sensors and key cards that turn the power on and off were added to the list. Kasim [81] showed that tourists were willing to accept rooms with energy-saving solutions.

In contrast, Zografakis et al. [82] found that hotel managers consider that tourists did not take into account hotel energy efficiency as a factor to select the hotel; only 53.2% agree or fully agree that tourists select their hotel accommodation based on its environmental image.

**Hypothesis 5** **(H5).** *Tourists’ attitude towards circular practices varies according to socio-economic profile: Nationality, educational level and income level.*

**Hypothesis 5a** **(H5a).** *Western tourists or tourists from rich countries have a more circular or pro-environmentally sustainable attitude than tourists from developing countries and Eastern and Asian tourists.*

Environmentally friendly hotel practices and nationality are correlated [44,51]. Baysan [53] suggested that German tourists seem to be more aware of the environmental consequences of tourism. Similarly, Lübbert [30] stated that about half of German tourists would consider an ecolabel when making travel decisions. Leonidou et al. [35] showed that tourists from Western European countries have a more environmentally friendly attitude than those from Eastern European countries. Finally, Barr [58] showed that nationality is a significant variable affecting the tourist attitude towards sustainability.

**Hypothesis 5b** **(H5b).** *There is a positive relationship between the tourists’ educational level and the circular or pro-environmentally sustainable attitude of tourists.*

Berezan et al. [44] suggested that environmentally friendly practices were significantly correlated with education. Environmental values and knowledge have significant and positive influences on the environmental behaviour intention of tourists [83]. In this sense, Dolnicar et al. [54] found that environmentally friendly tourists are higher-educated people with an interest in learning.

Leonidou et al., and Ayazlar and Gamze [35,47] evidenced that those tourists with higher educational levels have a more environmentally friendly attitude. Similarly, Ramchurjee and Suresha [55] demonstrated that tourists who have a bachelor’s degree and above (52.4%) had more environmentally friendly beliefs. Lita et al. [84] found that highly educated tourists have a more positive attitude and behaviour towards green practices. However, Chia-Jung and Pei-Chun [85] showed that having a higher level of education is associated with less green consumer behaviour. Similarly, Kollmuss and Agyeman [86] showed that more education does not necessarily mean increased pro-environmental behaviour.

**Hypothesis 5c** **(H5c).** *There is a positive relationship between the tourists’ income level and the circular or pro-environmentally sustainable attitude of tourists.*

According to Ayazlar and Gamze [47], previous studies indicated that customers who have an environmental conscience are more likely to gain more. Leonidou et al. and Dolnicar et al. [35,54] showed that those tourists who are higher-income earners have a more environmentally friendly attitude. Similarly, Chia-Jung and Pei-Chun [85] found that having a higher income is related to higher green consumer behaviour. The higher-income tourists were more willing to accept that personal toiletries were not provided in hotel rooms.

Similarly, Kasperson et al. [87] considered that tourists who accept the use of recycled water are characterised by having: High income, high levels of education and advanced age.

Kang et al. [68] argued that other studies obtained different results; in fact, Power and Elster [88], and UNDP [89] showed that low-income people are more sensitive towards the environment because their quality of life is more influenced by environmental topics.

**Hypothesis 6** **(H6).** *Recycling practices and reuse of towel and linen are the most common sustainable/circular hotel practices carried out by tourists.*

Reuse of towels or linens has received great attention in the literature [90,91,92]. In fact, the most common sustainable hotel practices that customers value more are the use of recycling bins and reusable towel and linen schemes [93,94,95].

Berezan et al. [44] stated that a towel reuse policy is one of the most widely recognised green practices, especially in the United States. Dimara et al. [96] also found that 72% of the tourists in hotels in two Greek cities would adopt a towel reuse program.

According to the Deloitte Consumer Survey [79], survey respondents indicated the following green initiatives as the most important ones: Recycling, energy-efficient lighting, energy-efficient windows, in-room cards for indicating the option of not having sheets/towels changed daily and environmentally safe cleaning products. Similarly, in a study by Kasim [81], tourists were willing to book rooms with water-saving technologies, recycling bins, energy-saving solutions, and information on local ecotourism attractions; and Andereck [38] stated that more than half of tourists believed that environmentally responsible initiatives, such as recycling bins or products made from recycled materials, were extremely important.

However, Tartaglia and de Grosbois [97] found out that the majority of respondents did not engage at all or only sometimes in the use of recycling bins, and the change of sheets and towels when necessary. Thus, tourists engaged strongly in energy and water conservation practices but not in the reuse of towels.

**Hypothesis 7** **(H7).** *There are gender differences in tourists’ circular practices in hotels.*

The environmental behaviour of tourists according to their gender has been extensively investigated. Several studies found that women tend to be more ecologically conscious than men [49,50].

According to Mensah [51], women were more environmentally responsible than men. They had a greater tendency to switch off lights when leaving their rooms, to purchase local souvenirs and food, to recycle correctly and not to buy things not needed. Laroche et al. [45] also proved that females were more environmentally conscious than males and were willing to pay more for green products. Similarly, Millar and Baloglu [80] discovered that preferences for green attributes were higher for females than for males on eight out of the twelve attributes analysed, and that is an indicator of a higher pro-environmental behaviour of females.

For Mensah [51], gender socialisation in women leads them to greater environmental sensitivity, and hence, to higher pro-environmental behaviours than men [98,99,100]. However, other studies have not established significant differences in the pro-environmental behaviours of males and females [101,102].

**Hypothesis 8** **(H8).** *There are differences between the circular practices carried out while on holidays in a hotel and those carried out at the tourist place of residence.*

There are studies that have investigated the differences between pro-environmental behaviour at home and on vacation. For example, Miao and Wei [103] showed that tourists’ active environmentalism while staying in a hotel is different from that in their household.

Other studies have shown that people tend to engage in pro-environmental behaviour at home more than when travelling [104,105]. For these authors, tourists are felt more morally obligated to behave in an environmentally sustainable manner at home than when on vacation. In a study by Baker et al. [74], about 60% of respondents recycled paper products at home while only 30% did so while at a hotel; 60% also conserved water at home and less than 40% did so at a hotel; and 80% of respondents conserved energy at home but only 40% who saved energy did so while staying at a hotel.

In addition, Dolnicar and Grün [106] stated that good environmental behaviour decreases during vacations compared to the home context. Similarly, Ramchurjee and Suresha [55] showed that tourists feel more responsible for the environment at home where they live and are willing to act in a more environmentally sustainable way in their immediate surroundings, and Holmes et al. [64] found that the more actions residents did at home, the more they would engage when traveling.

## 4. Materials and Methods

In order to analyse and answer the hypotheses raised, specific fieldwork was carried out through a structured questionnaire which combined open and closed questions. Face-to-face and online surveys followed a structured questionnaire divided into four sections, as can be seen in Table 1:

- First section focuses on travel characteristics such as type of hotel, board type, length of stay, travel purpose and degree of agreement with different circular economy-related statements, among others.
- Second section focuses on sustainable and/or circular practices carried out during the tourist’s stay at the hotel and on the island
- Third section focuses on sustainable and/or circular practices carried out at the tourist’s place of residence related to waste, water and energy consumption.
- Fourth section gathers information on the profile of the surveyed tourist.

Section 1 contains open and closed questions and one item rated on a five-point Likert scale that ranges from strongly disagree (1) to strongly agree (5). Section 2 contains questions rated on a Likert scale of 5 points, where 1 = strongly disagree and 5 = strongly agree, or questions with a Likert scale where 1 = never and 5 = always, and one open question about the incentives offered by the hotel to carry out environmentally sustainable practices. Section 3 includes one item rated on a Likert scale of 5 points where 1 = never and 5 = always and some open and closed questions. Finally, Section 4 contains open and closed questions related to the profile of the respondent.

### 4.1. Population

Gran Canaria received 4,267,385 tourists in 2019, of which 3,620,371 were foreign tourists [107]. Therefore, the population under study comprised tourists who visited Gran Canaria, and stayed at a hotel establishment. These tourists come from the main tourist-emitting countries: UK, Germany, Denmark, Finland, Norway, Sweden, the Netherlands, Italy, Belgium, France and Ireland; and also, national tourists arriving to the island in 2019 (in fact, they represent 15.16% of the total sample).

### 4.2. Sample Selection and Fieldwork

A total of 266 quantitative surveys, 211 face-to-face (79.32%) and 55 online (20.68%), containing 29 questions were collected considering the standard normal deviation set at 95% confidence level (1.96) and a margin of error ±6.009%. After information about the study was provided (i.e., topic, purpose and duration, confidentiality or language of the questionnaire), people were asked whether they were willing to take part in the study. Respondents were then provided with a structured face-to-face or online questionnaire designed in English, German and Spanish and conducted with tourists in multiple locations in Gran Canaria to ensure a random sample of tourists (tourists from different nationalities, ages, income level, staying at different hotels, etc.). Data were collected on the beach, in restaurants and on the promenade of the island’s main tourist areas from January to March 2020. For the face-to-face surveys, researchers were available to clarify meanings to ensure uniformity of responses as the respondents were of different nationalities. The survey took approximately 8–10 min to complete and consisted of 29 closed and open questions distributed in four sections.

Tourists were classified attending to several characteristics such as age, gender, occupation, educational level, nationality, residence country and income level. Table 2 shows the basic information of the tourists’ profile.

### 4.3. Analysis of the Data

After completing the fieldwork, data were tabulated using the SPSS statistical analysis programme (IBM, Madrid, Spain). Frequencies and correlation analysis were undertaken to obtain the tourist profile and to determine relationships and whether there were correlations between the different variables to be analysed.

## 5. Results

### 5.1. Validity and Reliability Analysis

To guarantee the scale’s validity, a great amount of literature from related studies has been revised to define concepts, ideas and issues under study, and the survey has been developed based on studies related to CE and tourism, the topic of this paper. Thus, the researchers were able to use an appropriate vocabulary in the questions, as well as to evaluate whether individual items appear to be appropriate measures of their respective constructs. On the other hand, the reliability of the scale’s items was carried out by using Cronbach’s alpha reliability coefficient that shows how well the items in a set are positively correlated to one another. Cronbach’s alpha values for each of the survey’s constructs are greater than 0.70 (Table 3), meaning all achieve an acceptable level, except the circular practices carried out during the tourists’ stay on the island with a value of 0.696. According to Sekaran and Bougie [108], reliabilities under 0.60 can be considered poor, while those in the 0.70 range can be considered acceptable. Additionally, the closer Cronbach’s alpha coefficient is to 1.0, the greater the internal consistency of the items in the scale, considering a 0.70 range acceptable and those over 0.80, good [109].

### 5.2. Tourist’s Profile

Descriptive statistics were performed to determine the tourist’s demographic characteristics. According to the results, the majority of participants are male (56%), more than 55 years old (54.1%), with university studies (44.7%), retired (43.6%) and with an income level between 2001 and 5000 Euros per month. The majority of respondents come from the United Kingdom (25.6%), Spain (16.5%) and Germany (12.8%) (See Table 2).

### 5.3. Travel Characteristics

Regarding travel characteristics, 57.9% of the respondents stayed at resort hotels while 30.1% stayed at urban city hotels. Most of the tourists (41.4%) stayed at a 3-star hotel. Taking into account the board type hired by the tourists, 47% of them had only room, 24.4% bed and breakfast, 17.7% half board and only 10.9% hired an all-inclusive board type.

The tourist’s length of stay on the island was 12 days on average in which 32% of them spent less than 500 Euros (meals, leisure, transportation, etc. included), 26.3% spent between 501 and 1000 Euros and 20.3% between 1001 and 2000 Euros.

When asked how they hired the hotel, 51.9% of tourists booked it online through platforms like Booking, TripAdvisor and Hotels.com, while 21.1% booked it through a Tour Operator like TUI or Condor. The rest of the respondents booked the hotel with a travel agency or on the hotel website (13.5% each).

The vast majority of tourists (89.1%) travel to the island for leisure purposes. Additionally, 54.5% of the respondents travel to Gran Canaria as a couple, 18% with friends, 15.8% travel alone, 10.9% with family and only 0.8% travel with co-workers.

About 63.9% of tourists do not know if the hotel where they stayed has any environmental quality management system or environmental certification, 25.6% do not remember and only 10.5% know whether the hotel has an environmental management system. In addition, 35.7% of those who know if the hotel has any environmental quality management system or environmental certification say it has ISO 14001, 35% do not know which certification, 14.3% Ecolabel, and EMAS and Cradle to Cradle 7.1% each.

Finally, when the tourists were asked if they received environmental information, such as an environmental badge or environmental certificate, when booking the hotel, 72.6% of them replied that they did not receive any information, 20.3% did not remember it and only 7.1% were offered environmental information when booking.

### 5.4. Hypotheses Testing

#### 5.4.1. Tourists’ Attitude and Behaviour towards Circular Economy Practices in Hotel Establishments

Tourists were asked to indicate their degree of agreement with different statements related to CE in hotel establishments on a 5-point scale ranging from 1 (totally disagree) to 5 (totally in agreement). Table 4 shows that the statements with the highest agreement were the importance of tourist accommodation to carry out a responsible policy with the environment, the importance that hotels manage the way in which water is used to reduce consumption and/or maximise its reuse, and the importance that tourist accommodation reduces the volume of waste through recycling, reuse of waste or the sale of waste to a third company (mean = 4.41, 4.35 and 4.3, respectively). The statements that attracted the greatest disagreements were the importance of a hotel having a recycling and waste management policy, the willingness to pay more for a hotel with better environmental quality and the willingness to use collaborative platforms during their holidays (mean = 3.65, 3.71 and 3.36, respectively).

**Hypothesis 1** **(H1).** *Older tourists have a more circular attitude than younger tourists.*

To test H1, an ANOVA test has been carried out and the sample was divided into 3 age groups: Between 18 and 35 years, 36 to 55. and over 55 years. Table 5 shows that there are significant differences between the different tourists’ age groups in terms of attitude towards circular practices. In general, tourists over 55 years old have a more circular attitude than younger tourists. In fact, there are significant differences among the tourists’ age groups when they choose tourist accommodations; older tourists tend to consider it important that the hotel has some environmental quality certification and uses renewable energy, a water and energy-saving policy, and that it provides training to staff in environmental issues. Additionally, for older tourists, reducing water consumption, using renewable energy and having a waste management policy in hotel establishments are very important. However, tourists between 18 and 35 years old have a more positive attitude to be willing to use collaborative platforms during their stay than older ones.

**Hypothesis 2** **(H2).** *Tourists are willing to pay more for environmentally friendly or green hotels. The older the tourist is, the greater the willingness to pay more.*

Results (Table 6) indicate that 60.9% of tourists fully agree or agree to pay more for a hotel with better environmental quality, while only 4.9% totally disagree. In addition, there are only significant differences between tourists’ age and nationality and the willingness to pay more to stay in an environmentally friendly or green hotel. Tourists over 55 years old and Swedish and German tourists are more willing to pay more than younger tourists and tourists from other nationalities.

**Hypothesis 3** **(H3).** *There is a positive relationship between hotel category and tourists’ awareness regarding circular economy practices.*

To test H3, tourists were divided according to 3 hotel category groups: 1 and 2 stars, 3 stars, and 4–5 stars. Results in Table 7 shows that there are no significant differences between hotel category and tourists’ awareness regarding circular economy practices. Only tourists who stay in 4- or 5-star hotels give greater importance to the recycling and waste management policy. The same result is obtained if we considered two groups (1–3 stars and 4–5 stars) or 5 groups according to stars.

**Hypothesis 4** **(H4).** *Most tourists believe that it is important for the hotel to have an energy-saving policy.*

To test this hypothesis, the questionnaire included a question where tourists were asked to rate the statement ‘For me it is important that a hotel has an energy saving policy’ (where 1 = strongly disagree and 5 = strongly agree); tourists showed a high level of agreement (mean = 4.2).

In order to analyse if there are differences in tourists’ belief of the importance of a hotel to have an energy-saving policy according to gender, we have carried out an independent-samples *t*-test (Table 8). As for age and nationality, an ANOVA test has been carried out (Table 9). Results show that there are no significant differences according to gender but there are differences at 10% between nationality and the belief that it is important for the hotel to have an energy-saving policy. Swedish and Spanish tourists are more aware of this aspect. However, there are significant differences at 5% by age; tourists over 55 years of age are those who give more importance to the hotel having an energy-saving policy, while tourists in the age group between 18 and 35 do not give it as much importance.

**Hypothesis 5** **(H5).** *Tourists’ attitude towards circular practices varies according to socio-economic profile: Nationality, educational level and income level.*

**Hypothesis 5a** **(H5a).** *Western tourists or tourists from rich countries have a more circular or pro-environmentally sustainable attitude than tourists from developing countries and Eastern and Asian tourists.*

**Hypothesis 5b** **(H5b).** *There is a positive relationship between the tourists’ educational level and the circular or pro-environmentally sustainable attitude of tourists.*

**Hypothesis 5c** **(H5c).** *There is a positive relationship between the tourists’ income level and the circular or pro-environmentally sustainable attitude of tourists.*

To test H5a, tourists have been divided into different groups based on their nationality: German, Spanish, UK, Swedish and other countries (mainly from Italy, Finland, Norway and The Netherlands). As can be seen in Table 10, there are significant differences between tourists’ nationality and their attitude towards the circular practices carried out by the hotel establishment. German and Swedish tourists show a more circular attitude towards the environmental practices carried out by hotels. On the contrary, Spanish tourists give it less importance.

Tourists were divided according to their educational level to test H5b, taking into account the following levels: No studies, primary education, obligatory secondary education, Upper-Secondary education and University studies. According to results (Table 10), there is a positive relationship between tourist educational level and tourist’s circular attitude; particularly, those tourists with higher educational level consider it important that the hotel establishment has some environmental quality certification, a recycling and waste management policy, and that they provide training in environmental issues to staff.

Finally, tourists were divided according to their income level to test H5c: Those who earn less than 2000 Euros per month, between 2001 and 5000 Euros and those who earn more than 5000. Results show that there are no significant differences between the circular attitudes of tourists according to their income level (Table 10). There are only significant differences in the importance that tourists give to hotels having recycling and waste management policies. Tourists with an income of more than 5000 Euros per month value this aspect more than the rest of tourists. On the other hand, there are significant differences at 10% in the importance that hotel staff have training in environmental issues (tourists who earn between 2001 and 5000 Euros per month give it greater importance) and the willingness to use collaborative platforms during the stay (tourists who earn less than 2000 Euros are more willing to use them).

**Hypothesis 6** **(H6).** *Recycling practices and reuse of towel and linen are the most common sustainable/circular hotel practices carried out by tourists.*

Tourists were asked how often they carried out different sustainable practices during their stay at the hotel and on the island based on a scale of 1–5; 1 = never, 2 = very rarely, 3 = sometimes, 4 = almost always, and 5 = always. Table 11 shows that the most common sustainable practices carried out during the tourist’s stay at the hotel are showering instead of bathing and recycling if the hotel has recycling bins (mean = 4.59 and 4.45, respectively), while the least carried out practices are: Not turning the air conditioner thermostat below 22 °C, and using the partial discharge tank (mean = 2.29 and 3.37, respectively). Asking for a change of sheets or towels only when necessary is also a common circular practice (mean = 4.21).

If we consider the sociodemographic profile, tourists’ behaviour towards recycling practices and reuse of towel and linen only varies according to gender and nationality. As for gender, women recycle if the hotel has recycling bins and ask for a change of sheets or towels only when necessary more than men. According to nationality, an ANOVA test has been carried out, and results indicate that tourists from Germany, Sweden and the United Kingdom ask for a change of sheets or towels only when necessary, while tourists from Spain and other countries ask for a change much more frequently.

**Hypothesis 7** **(H7).** *There are gender differences in tourists’ circular practices in hotels.*

The independent-samples *t*-test was carried to show if there are differences between the tourists’ circular practices in hotels according to gender. Table 12 shows that there are significant gender differences in some of the tourists’ circular practices in hotels. Specifically, women report a higher circular behaviour than men in not turning the air conditioning down below 22 °C, in recycling, trying to reduce food waste in restaurants and asking for a change of sheets or towels only when necessary.

Table 13 presents how often tourists carried out sustainable or circular practices during their stay in Gran Canaria. Results show that the circular practices they carry out more often are using reusable bags when buying and using public transport (mean = 4.18 and 3.97, respectively), while the practices they perform less frequently are participating in environmental recovery actions and carrying out environmentally sustainable leisure activities (hiking, bike routes, stargazing, visit natural parks, etc.) (mean = 2.37 and 3.00, respectively).

Additionally, tourists were asked if the hotel establishment where they stayed offered any type of incentives for the guest to carry out environmentally sustainable practices such as discounts for another stay, prizes, tickets to natural parks, etc. Results show that 90.6% of them did not receive any incentives while only 9.4% received it. The incentives that tourists received the most were tickets for leisure and natural parks (28%) and discounts for leisure activities (21%), while the least received incentives were beach cleaning activities (4%) and a basket of products without plastic (5%).

#### 5.4.2. CE Practices Carried Out at the Tourist’s Place of Residence

Tourists were asked to indicate how often they carry out different sustainable actions in their daily life in the country where they reside based on a scale of 1–5; 1 = never, 2 = very rarely, 3 = sometimes, 4 = almost always, and 5 = always. Practices were divided in three groups: Waste, water and energy and other types of sustainable actions. Table 14 shows that the most common sustainable waste-related practices carried out at the tourist’s place of residence are recycling paper and cardboard, and recycling plastic containers (both with a mean = 4.75), while the least carried out practices are buying/selling second-hand products (furniture, appliances, clothes, etc.) and keeping in mind that when buying clothes, it is environmentally sustainable (both with mean = 2.82). According to water and energy practices, the most common ones are trying to save water and energy and turning off air conditioning, heating and lights when leaving home (mean = 4.49 and 4.33, respectively), while the least carried out practices are using sustainable transport and using renewable energy sources (mean = 2.50 and 2.87, respectively). Finally, taking into account the other types of sustainable actions that tourists carry out at their place of residence, the most common one is promoting environmental awareness in the family (mean = 3.95) and the least carried out is using sharing platforms (mean = 2.80).

**Hypothesis 8** **(H8).** *There are differences between the circular practices carried out while on holidays in a hotel and those carried out at the tourists’ place of residence.*

**Figure 1:**
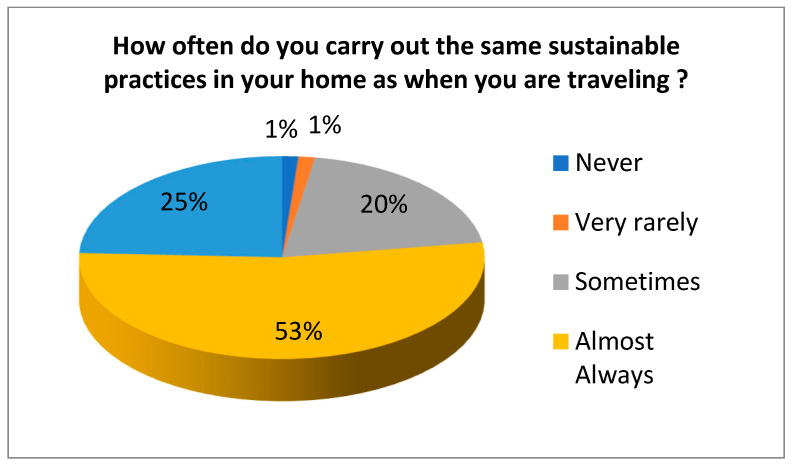
How often tourists carry out the same circular practices in their place of residence as when travelling.

The survey included a question related to H8 where tourists were asked to point out if they carry out the same circular practices in their place of residence as when travelling. Results indicate that 86.5% of tourists carry out the same environmentally sustainable practices. Additionally, tourists were asked to indicate how often they carry out the same circular practices in their place of residence as when travelling. Figure 1 shows that 53% carry out the same environmentally sustainable practices almost always and 24.3% do it always.

To test this hypothesis, an independent-samples *t*-test for gender and ANOVA test for age and nationality have been carried out. Results indicate that there are no significant differences by gender or nationality in the frequency of doing the same circular practices at home than when travelling; however, there are significant differences according to tourist’s age. Tourists over 55 do the same circular practices at home as when traveling more frequently than younger tourists. Hence, older tourists have the same environmental behaviour at home than when on holidays and younger tourists behave differently in their place of residence than when travelling.

## 6. Discussion

Our results support hypothesis H1 and are consistent with those of Leonidou et al., Ayazlar and Gamze, and Dolnicar [35,47,48], indicating that older tourists in Gran Canaria have a higher pro-environmental or circular attitude in hotel establishments than younger tourists.

Findings also indicate that most tourists (60.9%) are willing to pay more for environmentally friendly or green hotels. This result supports hypothesis H2, and confirms the results of Berezan et al., Masau and Prideaux, Kelly et al., and Han et al. [44,59,65,66]. Our results also show that tourists’ willingness to pay for more environmentally sustainable hotels is related to their socio-demographic profile. There are significant differences by tourists’ age or nationality in their willingness to pay more for a hotel that is environmentally responsive. The older a tourist is, the greater the willingness to pay more; this result is consistent with that of Mensah and Mensah [46] who pointed out that only age is significantly related to willingness to pay more. Furthermore, our results indicate that there is no gender or educational differences. However, Laroche et al. [45] proved that females were more environmentally conscious than males and were willing to pay more, and Mensah and Mensah [46] obtained that there were also strong relationships between the willingness to pay and the level of education.

On the other hand, our results show that there is no relationship between hotel category and tourists’ awareness regarding circular economy practices; therefore, hypothesis H3 is not supported. These results do not match with those of Kang et al. [68] who stated that luxury and mid-priced hotel guests are more willing to pay premiums for hotels’ green practices than guests of lower-category hotels.

Results also show that most tourists believe that it is important for the hotel to have an energy-saving policy, supporting hypothesis H4. The results are in line with those of Dalton et al. [78] and those of the Deloitte Consumer Survey [79], which identified energy-efficient lighting and energy-efficient windows as the most important green initiatives for tourists. However, they are opposite to those of Zografakis et al. [82] who argued that tourists do not consider hotel energy efficiency as a factor to select the hotel.

Additionally, our results indicate that tourists´ attitude towards circular practices varies according to socio-economic profile. First, there are significant differences between tourists’ nationality and their attitude towards the circular practices carried out by the hotel establishment; therefore, hypothesis H5a is supported. This result is consistent with those of Leonidou et al. and Berezan et al. [35,44,52] who showed that tourists from Western European countries have a more environmentally friendly attitude than those from Eastern European countries. Second, there is a positive relationship between tourist educational level and tourist’s circular attitude, so hypothesis H5b is also supported. These results are in line with Berezan et al. [44] who suggested that environmentally friendly practices were significantly correlated with education. In this sense, Dolnicar et al. [54] found that environmentally friendly tourists are people with higher educational levels and with an interest in learning. Similarly, Leonidou et al., and Ayazlar and Gamze [35,47] evidenced that those tourists with higher educational levels have a more environmentally friendly attitude. Third, results show that there are no significant differences between the circular attitudes of tourists according to their income level; therefore, the results do not support hypothesis H5c. Thus, these results do not match with the results of numerous authors such as Leonidou et al., Ayazlar and Gamze, Dolnicar et al., and Chia-Jung and Pei-Chun [35,47,54,85] who found that those tourists who are higher-income earners have a more environmentally friendly attitude.

On the other hand, findings also support hypothesis H6 and are consistent with those of Kim Lian Chan and Baum, Han and Kim, and Millar et al. [93,94,95] who stated that the most common sustainable hotel practices carried out by tourists are the use of recycling bins and reusable towel and linen schemes. Similarly, Berezan et al. [44] stated that one of the most widely recognised green practices, especially in the United States, is the towel reuse policy. Additionally, results show that tourists’ behaviour towards recycling practices and reuse of towel and linen only varies according to gender or nationality. Specifically, women recycle and ask for a change of sheets or towels only when necessary, while men change them more frequently, and tourists from Germany, Sweden and the United Kingdom ask for a change of sheets or towels only when necessary, while tourists from Spain and other countries are less aware and change them more often.

Additionally, women also report a higher circular behaviour than men in not turning the air conditioning down below 22 °C and trying to reduce food waste in restaurants. This result supports hypothesis H7 and is consistent with those of Mensah and Mensah, and Laroche et al. [45,46] who proved that women were more environmentally conscious than men. Similarly, Millar and Baloglu [80] found that preferences for green attributes were higher for women than for men on eight out of twelve attributes, indicating a higher pro-environmental behaviour of females.

Finally, this work investigated if there were differences between the circular practices carried out while on holidays in a hotel and those carried out at the tourist place of residence. Results indicate that 86.5% of tourists carry out the same environmentally sustainable practices; therefore, hypothesis H8 is not supported. However, if we analyse how often they carry out the same circular practices in their place of residence as when travelling according to socio-demographic factors, results indicate that there are no significant differences by gender or nationality in the frequency of doing the same circular practices at home as when travelling. However, there are significant differences according to tourists’ age. Tourists over 55 years old do the same circular practices at home as when traveling more frequently than younger tourists. These results do not match with those of Ramchurjee and Suresha, Dolnicar and Leisch, Miao and Wei, and Dolnicar and Grün [55,103,105,106] who found that good environmental behaviour decreases during vacations compared to the home context.

## 7. Conclusions

Tourism is an important contributor to economic growth, employment and GDP in many countries and regions, especially in the Canary Islands where it represents 35.2% of GDP and 40.2% of employment [3]. Nevertheless, tourism growth and development in the Canaries have altered the state of the coastal environment and have generated negative externalities on the environment. The implementation of CE models and solutions is especially important at island destinations to reduce environmental impacts generated by tourism activity. Tourism businesses and destinations can take advantage of many CE initiatives to reduce the trend but also to achieve greater profitability, increasing revenues in the provision of services, for example, in the hotel sector [21]. In this sense, the aim of this paper was to analyse the attitude towards CE and the environmental behaviour and circular practices among tourists of a well-known mature sun and beach destination, Gran Canaria, in order to design the transition from a linear model to a circular model in the hotel industry of this destination.

The first objective of the paper was to analyse the attitude of tourists towards CE in hotel establishments. The results indicate first that tourists’ attitude towards circular practices varies according to socio-economic profile: Nationality, educational level and income level. Specifically: German and Swedish tourists and those tourists with higher educational level show a more circular attitude towards the environmental practices carried out by hotels than the rest of the tourists. However, there are no significant differences in the circular attitudes of tourists according to their income level; there are only significant differences in the importance that tourists give to hotels having recycling and waste management policies. Second, older tourists have a higher pro-environmental or circular attitude in hotel establishments than younger ones. Findings also show that most tourists (60.9%) are willing to pay more for environmentally friendly or green hotels and believe that it is important for the hotel to have an energy-saving policy. Therefore, these results indicate which type of tourists, according to socio-economic profile, hotels and destinations, should place greater emphasis on conveying the message of the importance of having a circular attitude during their holidays. Hence, the objective was to identify the tourists who have a more circular attitude and behaviour at a mature destination (Gran Canaria) according to their socio-economic profile.

The second objective was to analyse the environmental behaviour and circular practices carried out by tourists. Results show that the most common sustainable hotel practices carried out by tourists are the use of recycling bins and reusable towel and linen schemes, and that women report significantly higher pro-environmental behaviours than men. Consequently, hotel managers should consider having recycling bins in their establishments and guests should be offered information about the change of towels and sheets only when necessary as these two aspects are quite well received by tourists

Furthermore, results indicate that 86.5% of tourists carry out the same CE practices on holidays as in their place of residence and there are only significant differences according to tourists’ age in the frequency of doing the same circular practices at home as when travelling. Tourists over 55 years old carry out more frequently the same circular practices at home as when traveling than younger tourists. Additionally, tourists were asked about the circular practices they carry out at their place of residence, and findings show that the most common practices are recycling paper and cardboard and plastic containers, trying to save water and energy, and turning off air conditioning, heating and lights when leaving home. Destinations and hotels should take this into account to promote these types of practices during the tourist’s stay.

The COVID-19 pandemic has obviously enormous negative economic consequences in the tourism sector, especially at island destinations such as Gran Canaria, but it also poses challenges and opens up new opportunities for the tourism sector. It has shown that tourist businesses need to be flexible and ready for change. Many businesses will look for an increase in brand image while reducing the cost associated, and them moving away from a linear economy model towards the CE in tourism is the possible solution [110]. As Zhang and Tian [111] stated, in order to increase the competitiveness of the tourism industry, circular tourism must be the solution. Therefore, environmental information and education by hotels to their guests are of great importance in order to achieve a change in the behaviour of tourists with respect to the CE. The tourism industry should focus on investing in training, innovation, analysis, research and resources to achieve the transition to a CE model in the sector.

To sum up, more research is needed on how to generate CE solutions towards a more environmentally sustainable tourism industry. Therefore, future research could focus on defining CE strategies and initiatives for hotels, tourism business and destinations to attract tourists who are more aware about the CE issue. Another future line of research could be the investigation of circular practices carried out by hotels and those that have to be implemented or promoted to achieve the change to a circular model in the tourism industry.

Finally, this study faces various limitations that could reduce the generalisation of its results. On the one hand, the study analyses the attitude towards CE and the environmental behaviour and circular practices among tourists of a well-known mature sun and beach destination, Gran Canaria. Thus, further empirical studies should be carried out for results to be representative for all sun and beach tourist destinations. On the other hand, results may be different at other types of tourist destinations, such as an urban or rural tourist destination; future research may involve this type of destinations and results comparison.

## Figures and Tables

**Table 1:** Structure of the questionnaire.

Section	Objective
I. Travel characteristics	Collects information about hotel type, board type, length of stay, travel purpose, total expenditure during the holiday, etc.
II. Sustainable practices at the hotel and on the island	Identifies the sustainable practices carried out by tourists during their stay at the hotel and on the island and collects information about degree of agreement with different circular economy-related statements.
III. Sustainable practices at home	Collects information about sustainable practices carried out at the tourist’s place of residence (waste, water, energy, etc.).
IV. Profile of the respondent	Checks the profile of the respondent (age, gender, occupation, educational level, nationality, income level, etc.).

**Table 2:** Tourists’ profile.

Total Number of Tourists/Percentage		
Age	From 18 to 35	48/18
	From 36 to 55	74/27.8
	More than 55	144/54.1
Gender	Male	149/56
	Female	117/44
Nationality	Germany	34/12.8
	Spain	44/16.5
	United Kingdom	68/25.6
	Sweden	27/10.2
	Others	93/35
Occupation	Self-employed entrepreneur	29/10.9
	Senior management employee, etc.	34/12.8
	Middle management employee	62/23.3
	Employee with no qualification	11/4.1
	Student	7/2.6
	Retired	116/43.6
	Unemployed	2/0.8
	Others	5/1.9
Educational level	No studies	7/2.6
	Primary education	28/10.5
	Obligatory secondary education	33/12.4
	Upper-Secondary education	67/25.2
	University studies	119/44.7
	Prefer not to answer	12/4.5
Income level	Less than 2000 €	36/13.5
	2001–5000 €	138/51.9
	More than 5000 €	47/17.7
	NR/DK	45/16.9

**Table 3:** Reliability analysis and descriptive statistics (N = 266).

Construct	No. of Items	Cronbach´s Alpha	Mean	Variance
Tourists’ attitude towards circular economy practices in hotel establishments	13	0.907	4.034	0.108
Circular practices at the hotel	7	0.773	4.012	0.382
Circular practices on the island	7	0.696	3.5	0.390
Circular practices at the place of residence	25	0.888	3.745	0.484

**Table 4:** Statements related to circular economy (CE) in hotel establishments.

Statements	N	Mean
For me it is important that tourist accommodation carry out a responsible policy with the environment	266	4.41
When I choose a tourist accommodation, for me it is important that it has some environmental quality certification	266	3.73
I am willing to pay more for a hotel with better environmental quality	266	3.71
For me it is important that a hotel has a recycling and waste management policy	266	3.65
For me it is important that a hotel has a water saving policy	266	4.2
For me it is important that a hotel has an energy saving policy	266	4.2
For me it is important that hotel staff have training in environmental issues (recycling, etc.)	266	4.22
For me it is important that hotels manage the way in which water is used to reduce consumption and/or maximize its reuse	266	4.35
For me it is important that tourist accommodation use renewable energy	266	4.15
For me it is important that tourist accommodation manage the use and consumption of energy to minimize its consumption.	266	4.29
I am willing that hotels offer closed menus in restaurants to reduce food waste	266	3.88
For me it is important that tourist accommodation reduce the volume of waste through recycling, reuse of waste or the sale of waste to a third company.	266	4.3
I would be willing to use collaborative platforms during my stay (BlaBlaCar, foodtogo, etc.) (if possible)	266	3.36

**Table 5:** ANOVA test comparing attitude towards circular practices by age groups.

	df	Mean	F	Sig.
For me it is important that tourist accommodation carry out a responsible policy with the environment	2	4.41	2.022	0.134
When I choose a tourist accommodation, for me it is important that it has some environmental quality certification	2	3.73	12.020	0.000
For me it is important that a hotel has a recycling and waste management policy	2	3.65	0.450	0.638
For me it is important that a hotel has a water saving policy	2	4.2	6.592	0.002
For me it is important that a hotel has an energy saving policy	2	4.2	7.406	0.001
For me it is important that hotel staff have training in environmental issues (recycling, etc.)	2	4.22	7.345	0.001
For me it is important that hotels manage the way in which water is used to reduce consumption and/or maximize its reuse	2	4.35	6.649	0.002
For me it is important that tourist accommodation use renewable energy	2	4.15	5.826	0.003
For me it is important that tourist accommodation manage the use and consumption of energy to minimize its consumption.	2	4.29	6.267	0.002
I am willing that hotels offer closed menus in restaurants to reduce food waste	2	3.88	3.178	0.043
For me it is important that tourist accommodation reduce the volume of waste through recycling, reuse of waste or the sale of waste to a third company.	2	4.3	3.892	0.022
I would be willing to use collaborative platforms during my stay (BlaBlaCar, foodtogo, etc.) (if possible)	2	3.36	0.645	0.525

**Table 6:** Willingness to pay more for environmentally friendly or green hotels.

	Frequency	Percentage	Accumulated Percentage
Totally disagree	13	4.9	4.9
2.00	15	5.6	10.5
3.00	76	28.6	39.1
4.00	94	35.3	74.4
Totally in agreement	68	25.6	100.0
Total	266	100.0	

**Table 7:** ANOVA test comparing attitude towards circular practices by hotel category groups.

	df	Mean	F	Sig.
For me it is important that hotels carry out a responsible policy with the environment	2	4.41	0.008	0.992
When I choose a tourist accommodation, for me it is important that it has some environmental quality certification	2	3.73	0.106	0.899
For me it is important that a hotel has a recycling and waste management policy	2	3.65	3.900	0.021
For me it is important that a hotel has a water saving policy	2	4.2	0.498	0.608
For me it is important that a hotel has an energy saving policy	2	4.2	0.182	0.834
For me it is important that hotel staff have training in environmental issues (recycling, etc.)	2	4.22	0.481	0.619
For me it is important that hotels manage the way in which water is used to reduce consumption and/or maximize its reuse	2	4.35	0.155	0.857
For me it is important that tourist accommodation use renewable energy	2	4.15	0.148	0.863
For me it is important that tourist accommodation manage the use and consumption of energy to minimize its consumption.	2	4.29	0.456	0.634
I am willing that hotels offer closed menus in restaurants to reduce food waste	2	3.88	0.261	0.770
For me it is important that tourist accommodation reduce the volume of waste through recycling, reuse of waste or the sale of waste to a third company	2	4.3	0.286	0.752
I would be willing to use collaborative platforms during my stay (BlaBlaCar, foodtogo, etc.) (if possible)	2	3.36	0.146	0.864

**Table 8:** *t*-Test comparing the importance for the hotel to have an energy-saving policy by gender.

Levene’s Test for Equality of Variances				*t*-Test for Equality of Means						
F	Sig.			t	df	Sig.	Mean Difference	Std. Error Difference	95% Confidence Interval of the Difference	
									Lower	Upper
For me it is important that a hotel has an energy saving policy	Equal variances assumed	2.815	0.095	−0.949	264	0.343	−0.10876	0.11456	−0.33432	0.11680
	Equal variances not assumed			−0.935	232.448	0.351	−0.10876	0.11634	−0.33798	0.12046

**Table 9:** ANOVA test comparing the importance for the hotel to have an energy-saving policy by nationality or age.

		df	Mean	F	Sig.
For me it is important that a hotel has an energy saving policy	**Nationality**	4	4.2	2.195	0.070
	**Age**	2	4.2	7.406	0.001

**Table 10:** ANOVA test comparing tourists’ attitude towards circular practices according to socio-economic profile.

		df	Mean	F	Sig.
For me it is important that tourist accommodation carry out a responsible policy with the environment	Nationality	4	4.41	5.283	0.000
	Educational level	5	4.41	0.703	0.621
	Income level	3	4.41	0.808	0.490
When I choose a tourist accommodation, for me it is important that it has some environmental quality certification	Nationality	4	3.73	2.183	0.071
	Educational level	5	3.73	2.518	0.030
	Income level	3	3.73	1.270	0.285
For me it is important that a hotel has a recycling and waste management policy	Nationality	4	3.65	26.137	0.000
	Educational level	5	3.65	4.758	0.000
	Income level	3	3.65	9.264	0.000
For me it is important that a hotel has a water saving policy	Nationality	4	4.2	1.703	0.150
	Educational level	5	4.2	1.465	0.202
	Income level	3	4.2	1.096	0.351
For me it is important that a hotel has an energy saving policy	Nationality	4	4.2	2.195	0.070
	Educational level	5	4.2	0.953	0.447
	Income level	3	4.2	0.465	0.707
For me it is important that hotel staff have training in environmental issues (recycling, etc.)	Nationality	4	4.22	2.081	0.084
	Educational level	5	4.22	2.321	0.044
	Income level	3	4.22	2.621	0.051
For me it is important that hotels manage the way in which water is used to reduce consumption and/or maximize its reuse	Nationality	4	4.35	4.049	0.003
	Educational level	5	4.35	0.694	0.628
	Income level	3	4.35	0.826	0.481
For me it is important that tourist accommodation use renewable energy	Nationality	4	4.15	5.120	0.001
	Educational level	5	4.15	1.213	0.303
	Income level	3	4.15	1.004	0.392
For me it is important that tourist accommodation manage the use and consumption of energy to minimize its consumption.	Nationality	4	4.29	3.166	0.015
	Educational level	5	4.29	0.603	0.697
	Income level	3	4.29	1.022	0.383
I am willing that hotels offer closed menus in restaurants to reduce food waste	Nationality	4	3.88	6.192	0.000
	Educational level	5	3.88	1.402	0.224
	Income level	3	3.88	0.856	0.465
For me it is important that tourist accommodation reduce the volume of waste through recycling, reuse of waste or the sale of waste to a third company.	Nationality	4	4.3	2.058	0.087
	Educational level	5	4.3	0.861	0.508
	Income level	3	4.3	1.955	0.121
I would be willing to use collaborative platforms during my stay (BlaBlaCar, foodtogo, etc.) (if possible)	Nationality	4	3.36	6.700	0.000
	Educational level	5	3.36	1.164	0.327
	Income level	3	3.36	2.118	0.098

**Table 11:** Circular practices carried out by tourists at the hotel.

Circular Practices at the Hotel	N	Mean
I shower instead of bathing	266	4.59
I use the partial discharge tank	266	3.37
I turn off air conditioning and lights when I leave the room	266	4.29
Not turning the air conditioner thermostat below 22 °C	266	2.29
Recycle if the hotel has recycling bins	266	4.45
I try to reduce food waste in restaurants	266	4.26
I ask for a change of sheets or towels only when necessary	266	4.21

**Table 12:** ANOVA test comparing tourists’ circular practices in hotels by gender.

	df	Mean	F	Sig.
I shower instead of bathing	1	4.59	2.154	0.143
I use the partial discharge tank	1	3.37	1.738	0.189
I turn off air conditioning and lights when leaving the room	1	4.29	2.618	0.107
Not turning the air conditioner thermostat below 22 °C	1	2.92	4.292	0.039
Recycle if the hotel has recycling bins	1	4.45	4.942	0.027
I try to reduce food waste in restaurants	1	4.26	8.111	0.005
I ask for a change of sheets or towels only when necessary	1	4.21	10.862	0.001

**Table 13:** Circular practices carried out by tourists on the island.

Circular Practices on the Island	N	Mean
I use public transport	266	3.97
I buy in small shops instead of big chains	266	3.68
I buy products with less packaging	266	3.82
I use reusable bags when buying	266	4.18
I carry out environmentally sustainable leisure activities (hiking, bike routes, stargazing, visit natural parks...)	266	3.00
I participate in environmental recovery actions (beach cleaning, tree planting...)	266	2.37
I am interested in knowing the environment of the island and its environmental problems	266	3.48

**Table 14:** Circular practices carried out at the tourist’s place of residence.

Sustainable Practices at the Place of Residence (Waste)	N	Mean
I recycle glass	266	4.71
I recycle paper and cardboard	266	4.75
I recycle cooking oil	266	3.58
I recycle plastic containers	266	4.75
I recycle household appliances, printers, computers...	266	4.25
I separate the organic waste	266	4.09
I avoid wasting food	266	4.36
I try to repair before buying	266	3.95
I buy/sell second-hand products (furniture, appliances, clothes, etc.)	266	2.82
When I buy clothes I notice that it is environmentally sustainable	266	2.82
I buy local and/or seasonal products	266	3.77
I buy organic products	266	3.07
I buy products with less packaging	266	3.84
I use reusable bags when buying	266	4.41
I avoid aluminium foil	266	3.10
Sustainable practices at the place of residence (Water and energy)		Mean
I try to save water and energy	266	4.49
I use renewable energy sources	266	2.87
I use public transport	266	3.70
I use sustainable transport (bicycle, electric car, etc.)	266	2.50
I turn off air conditioning, heating and lights when I leave home	266	4.33
Thermostat with time programming	266	3.76
I take into account the level of energy efficiency in the house	266	3.97
Sustainable practices at the place of residence (Other)	N	Mean
I do environmentally sustainable leisure activities (hiking, cycling, stargazing)	266	2.98
In the family we promote environmental awareness	266	3.95
I use shared platforms (if any).	266	2.80

## References

[1] Ellen MacArthur Foundation Towards a Circular Economy: Business Rationale for an Accelerated Transition. https://www.ellenmacarthurfoundation.org/.

[2] Jacob M., Florido C., Aguiló E. (2010). Research note: Environmental innovation as a competitiveness factor in the Balearic Islands. Tour. Econ..

[3] Exceltur (2018). Impactur Canarias. https://www.exceltur.org/wp-content/uploads/2019/12/IMPACTUR-Canarias-2018.pdf.

[4] Hunter C., Green H. (1995). Tourism and the Environment: A Sustainable Relationship?.

[5] Florido C., Jacob M., Payeras M. (2019). How to Carry out the Transition towards a More Circular Tourist Activity in the Hotel Sector. The Role of Innovation. Adm. Sci..

[6] Pulido-Fernández J.I., López-Sánchez Y. (2016). Are tourists really willing to pay more for sustainable destinations?. Sustainability.

[7] Smol M., Avdiushchenko A., Kulczycka J., Nowaczek A. (2018). Public awareness of circular economy in southern Poland: Case of the Malopolska region. J. Clean. Prod..

[8] Halpenny E.A. (2010). Pro-environmental behaviours and park visitors: The effect of place attachment. J. Environ. Psychol..

[9] Sørensen F., Bærenholdt J.O. (2020). Tourist practices in the circular economy. Ann. Tour. Res..

[10] Nedyalkova S. Applying circular economy principles to sustainable tourism development. Proceedings of the PM4SD European Summer School-Abstract and Conference Proceedings.

[11] Giurea R., Precazzini I., Ragazzi M., Achim M.I., Cioca L.-I., Conti F., Torretta V., Rada E.C. (2018). Good Practices and Actions for Sustainable Municipal Solid Waste Management in the Tourist Sector. Resources.

[12] Stein N., Spinler S., Vanthournout H., Blass V. (2020). Consumer perception of online attributes in circular economy activities. Sustainability.

[13] Giurea R. (2018). Contributions Regarding the Research of the Sustainable Development in Agro-Tourism from a Circular Economy Perspective. Ph.D. Thesis.

[14] European Commission The Circular Economy-Connecting, Creating and Conserving Value. https://www.eesc.europa.eu/resources/docs/the-circular-economy.pdf.

[15] Domenech T., Bahn-Walkowiak B. (2019). Transition towards a resource efficient circular economy in Europe: Policy lessons from the EU and the member states. Ecol. Econ..

[16] European Commission A New Circular Economy Action Plan. For a Cleaner and More Competitive Europe. https://eur-lex.europa.eu/resource.html?uri=cellar:9903b325-6388-11ea-b735-01aa75ed71a1.0017.02/DOC_1&format=PDF.

[17] European Commission Closing the Loop- an EU Action Plan for the Circular Economy. https://ec.europa.eu/transparency/regdoc/rep/1/2015/EN/1-2015-614-EN-F1-1.PDF.

[18] EEA (2010). European Environment, State and Outlook 2010.

[19] United Nations Environment Programme A Manual for Water and Waste Management: What the Tourism Industry Can Do to Improve its Performance. https://www.unep.org/.

[20] González Camazón C. (2016). La Fiscalidad Verde. Creación de Nuevos Tributos Sobre Emisiones y Residuos. Bachelor’s Thesis.

[21] Rodríguez C., Florido C., Jacob M. (2020). Circular Economy Contributions to the Tourism Sector: A Critical Literature Review. Sustainability.

[22] González M., León C. (2001). The adoption of environmental innovations in the hotel industry of Gran Canaria. Tour. Econ..

[23] Manniche J., Larsen K.T., Broegaard R.B., Holland E. (2017). Destination: A Circular Tourism Economy a Handbook for Transitioning toward a Circular Economy within the Tourism and Hospitality Sectors in the South Baltic Region.

[24] Pang S.F., McKercher B., Prideaux B. (2013). Climate change and tourism: An overview. Asia Pac. J. Tour. Res..

[25] Juvan E., Dolnicar S. (2016). Measuring environmentally sustainable tourist behavior. Ann. Tour. Res..

[26] Sørensen F., Bærenholdt J.O., Greve K.A.G.M. (2019). Circular economy tourist practices. Curr. Issues Tour..

[27] Alemão C.P. (2019). Environmental Sustainability Practices in the Supply Chain of Hotels and the Consumers’ Perception. Ph.D. Thesis.

[28] Goodwin H., Francis J. (2003). Ethical and responsible tourism: Consumer trends in the UK. J. Vacat. Mark..

[29] Jacobsen J.K.S. (2007). Monitoring motoring: A study of tourists’ viewpoints of environmental performance and protection practices. Scand. J. Hosp. Tour..

[30] Lübbert C., Font X., Buckley R.C. (2001). Tourism Ecolabels Market Research in Germany. Tourism Ecolabelling. Certification and Promotion of Sustainable Management.

[31] Puhakka R. (2011). Environmental concern and responsibility among nature tourists in Oulanka PAN Park, Finland. Scand. J. Hosp. Tour..

[32] Horner S., Swarbrooke J. (2016). Consumer Behaviour in Tourism.

[33] Hedlund T. (2011). The impact of values, environmental concern and willingness to accept economic sacrifices to protect the environment on tourists’ intentions to buy ecologically sustainable tourism alternatives. Tour. Hosp. Res..

[34] Kim Y., Choi S.M. (2005). Antecedents of green purchase behavior: An examination of collectivism, environmental concern, and PCE. Adv. Consum. Res..

[35] Leonidou L.C., Coudounaris D.N., Kvasova O., Christodoulides P. (2015). Drivers and outcomes of green tourist attitudes and behavior: Sociodemographic moderating effects. Psychol. Mark..

[36] Han H., Kim W., Kiatkawsin K. (2017). Emerging youth tourism; fostering Young travelers’ conservation intentions. J. Trav. Tour. Mark..

[37] Kvasova O. (2015). The big five personality traits as antecedents of eco-friendly tourist behavior. Person. Individ. Differ..

[38] Andereck K.L. (2009). Tourists’ perceptions of environmentally responsible innovations at tourism businesses. J. Sustain. Tour..

[39] Han H., Hyun S.S. (2018). What influences water conservation and towel reuse practices of hotel guests?. Tour. Manag..

[40] Robinot E., Giannelloni J.L. (2010). Do hotels’ “green” attributes contribute to customer satisfaction?. J. Serv. Mark..

[41] Powell R.B., Ham S.H. (2008). Can ecotourism interpretation really lead to pro-conservation knowledge, attitudes and behaviour? Evidence from the Galapagos Islands. J. Sustain. Tour..

[42] Tisdell C., Wilson C. (2005). Perceived impacts of ecotourism on environmental learning and conservation: Turtle watching as a case study. Environ. Dev. Sustain..

[43] Lee W.H., Moscardo G. (2005). Understanding the impact of ecotourism resort experiences on tourists’ environmental attitudes and behavioural intentions. J. Sustain. Tour..

[44] Berezan O., Millar M., Raab C. (2014). Sustainable hotel practices and guest satisfaction levels. Int. J. Hosp. Tour. Admin..

[45] Laroche M., Bergeron J., Barbaro-Forleo G. (2001). Targeting consumers who are willing to pay more for environmentally friendly products. J. Consum. Mark..

[46] Mensah I., Mensah R.D. (2013). International tourists’ environmental attitude towards hotels in Accra. Int. J. Acad. Res. Bus. Soc. Sci..

[47] Ayazlar A.P.D.R.A., Gamze G.Ü.N. The Attitudes of Customers towards Green Hotels. Proceedings of the 1st International Sustainable Tourism Congress.

[48] Dolnicar S. (2010). Identifying tourists with smaller environmental footprints. J. Sustain. Tour..

[49] McIntyre G., Inskeep E., Hetherington A. (1993). Desarrollo Turístico Sostenible: Guía Para Planificadores Locales.

[50] Banerjee B., McKeage K. (1994). How green is my value: Exploring the relationship between environmentalism and materialism. ACR N. Am. Adv..

[51] Mensah I. (2012). Environmental education and environmentally responsible behavior: The case of international tourists in Accra hotels. Int. J. Tour. Sci..

[52] Berezan O., Raab C., Yoo M., Love C. (2013). Sustainable hotel practices and nationality: The impact on guest satisfaction and guest intention to return. Int. J. Hosp. Manag..

[53] Baysan S. (2001). Perceptions of the environmental impacts of tourism: A comparative study of the attitudes of German, Russian and Turkish tourists in Kemer, Antalya. Tour. Geogr..

[54] Dolnicar S., Crouch G.I., Long P. (2008). Environment-friendly tourists: What do we really know about them?. J. Sustain. Tour..

[55] Ramchurjee N.A., Suresha S. (2015). Are tourists’ environmental behavior affected by their environmental perceptions and beliefs?. J. Environ. Tour. Anal..

[56] Paulraj A. (2009). Environmental motivations: A classification scheme and its impact on environmental strategies and practices. Bus. Strategy Environ..

[57] Perkins H.E., Brown P.R. (2012). Environmental values and the so-called true ecotourist. J. Travel Res..

[58] Barr S. (2007). Factors influencing environmental attitudes and behaviours: A UK case study of household waste management. Environ. Behav..

[59] Han H., Hsu L.-T., Lee J.-S. (2009). Empirical investigation of the roles of attitudes toward green behaviours, overall image, gender, and age in hotel customers’ eco-friendly decision-making process. Int. J. Hosp. Manag..

[60] Samdahl D.M., Robertson R. (1989). Social determinants of environmental concern: Specification and test of the model. Environ. Behav..

[61] Vining J., Ebreo A. (1990). What makes a recycler? A comparison of recyclers and nonrecyclers. Environ. Behav..

[62] Roberts J.A. (1996). Green consumers in the 1990s: Profile and Implications for Advertising. J. Bus. Res..

[63] Kiatkawsin K., Han H. (2017). Young travelers′ intention to behave pro-environmentally: Merging the value-belief-norm theory and the expectancy theory. Tour. Manag..

[64] Holmes M.R., Dodds R., Frochot I. (2019). At home or abroad, does our behavior change? Examining how everyday behavior influences sustainable travel behavior and tourist clusters. J. Travel Res..

[65] Masau P., Prideaux B. (2003). Sustainable Tourism: A Role for Kenya’s Hotel Industry. Curr. Issues Tour..

[66] Kelly J., Haider W., Williams P.W., Englund K. (2007). Stated preferences of tourists for eco-efficient destination planning options. Tour. Manag..

[67] Bohdanowicz P. (2003). A Study of Environmental Impacts, Environmental Awareness and Pro-Ecological Initiatives in the Hotel Industry. Ph.D. Thesis.

[68] Kang K.H., Stein L., Heo C.Y., Lee S. (2012). Consumers’ willingness to pay for green initiatives of the hotel industry. Int. J. Hosp. Manag..

[69] Borden D.S., Coles T., Shaw G. (2017). Social marketing, sustainable tourism, and small/medium size tourism enterprises: Challenges and opportunities for changing guest behaviour. J. Sustain. Tour..

[70] Kostakis I., Sardianou E. (2012). Which factors affect the willingness of tourists to pay for renewable energy?. Renew. Energy.

[71] Wehrli R., Schwarz J., Stettler J. (2011). Are tourists willing to pay more for sustainable tourism? A choice experiment in Switzerland. https://www.hslu.ch/de-ch/wirtschaft/institute/itw/working-paper-series/.

[72] Jauhari V., Manaktola K. (2007). Exploring consumer attitude and behaviour towards green practices in the lodging industry in India. Int. J. Contemp. Hosp. Manag..

[73] Lee J.-S., Hsu L.T., Han H., Kim Y. (2010). Understanding how consumers view green hotels: How a hotel’s green image can influence behavioural intentions. J. Sustain. Tour..

[74] Baker M.A., Davis E.A., Weaver P.A. (2014). Eco-friendly attitudes, barriers to participation, and differences in behavior at green hotels. Cornell Hosp. Q..

[75] Dimara E., Manganari E., Skuras D. Consumers’ willingness to pay premium for green hotels: Fact or Fad. Proceedings of the International Marketing Trends Conference.

[76] Mensah I. (2006). Environmental management practices among hotels in the greater Accra region. Int. J. Hosp. Manag..

[77] Ma X., Li S., Ai Q., Chen K. Research on renewable energy systems used in tourism circular economy. Proceedings of the 2016 Chinese Control and Decision Conference (CCDC).

[78] Dalton G.J., Lockington D.A., Baldock T.E. (2008). A survey of tourist attitudes to renewable energy supply in Australian hotel accommodation. Renew. Energy.

[79] (2008). Deloitte Consumer Survey. The Staying Power of Sustainability. https://www.expoknews.com/wp-content/uploads/2010/02/us_cb_sustainability_1906081.pdf.

[80] Millar M., Baloglu S. (2011). Hotel guests’ preferences for green guest room attributes. Cornell Hosp. Q..

[81] Kasim A. (2004). Socio-environmentally responsible hotel business: Do tourists to Penang Island, Malaysia care?. J. Hosp. Leis. Mark..

[82] Zografakis N., Gillas K., Pollaki A., Profylienou M., Bounialetou F., Tsagarakis K.P. (2011). Assessment of practices and technologies of energy saving and renewable energy sources in hotels in Crete. Renew. Energy.

[83] Rodríguez-Oromendía A., Reina-Paz M.D., Sevilla-Sevilla C. (2013). Environmental awareness of tourists. Environ. Eng. Manag. J..

[84] Lita R.P., Surya S., Ma’Ruf M., Syahrul L. (2014). Green attitude and behavior of local tourists towards hotels and restaurants in West Sumatra, Indonesia. Procedia Environ. Sci..

[85] Chia-Jung C., Pei-Chun C. (2014). Preferences and willingness to pay for green hotel attributes in tourist choice behavior: The case of Taiwan. J. Trav. Tour. Mark..

[86] Kollmuss A., Agyeman J. (2002). Mind the gap: Why do people act environmentally and what are the barriers to pro-environmental behavior?. Environ. Educ. Res..

[87] Kasperson R.E., Baumann B., Dworkin D., McCauley D., Reynolds J., Sims J.H. (1974). Community Adoption Water Reuse System in the United States.

[88] Power A., Elster J. (2005). Environmental Issues and Human Behaviour in Low-Income Areas in the UK.

[89] UNDP Human Development Report 2006: Beyond Scarcity: Power, Poverty and the Global Water Crisis, New York: UNDP. https://www.undp.org/content/undp/en/home/librarypage/hdr/human-development-report-2006.html.

[90] Goldstein N.J., Cialdini R.B., Griskevicius V. (2008). A room with a viewpoint: Using social norms to motivate environmental conservation in hotels. J. Consum. Res..

[91] Mair J., Bergin-Seers S. (2010). The effect of interventions on the environmental behaviour of Australian motel guests. Tour. Hosp. Res..

[92] Shang J., Basil D.Z., Wymer W. (2010). Using social marketing to enhance hotel reuse programs. J. Bus. Res..

[93] Kim Lian Chan J., Baum T. (2007). Motivation factors of ecotourists in ecolodge accommodation: The push and pull factors. Asia Pac. J. Tour. Res..

[94] Han H., Kim Y. (2010). An investigation of green hotel customers’ decision formation: Developing an extended model of the theory of planned behavior. Int. J. Hosp. Manag..

[95] Millar M., Mayer K.J., Baloglu S. (2012). Importance of green hotel attributes to business and leisure travelers. J. Hosp. Mark. Manag..

[96] Dimara E., Manganari E., Skuras D. (2017). Don´t change my towels please: Factors influencing participation in towel reuse programs. Tour. Manag..

[97] Tartaglia S., De Grosbois D. Comparison of tourists’ environmental beliefs and environmental behaviour. Proceedings of the Administrative Sciences Association Canada.

[98] Karpiak C.P., Baril G.L. (2008). Moral reasoning and concern for the environment. J. Environ. Psychol..

[99] Mostafa M.M. (2007). Gender differences in Egyptian consumers’ green purchase behaviour: The effects of environmental knowledge, concern and attitude. Int. J. Consum. Stud..

[100] Zelezny L.C., Chua P.P., Aldrich C. (2000). New ways of thinking about environmentalism: Elaborating on gender differences in environmentalism. J. Soc. Issues.

[101] Clark C.F., Kotchen M.J., Moore M.R. (2003). Internal and external influences on pro-environmental behavior: Participation in a green electricity program. J. Environ. Psychol..

[102] Tindall D.B., Davies S., Mauboules C. (2003). Activism and conservation behavior in an environmental movement: The contradictory effects of gender. Soc. Nat. Resour..

[103] Miao L., Wei W. (2013). Consumers’ pro-environmental behaviour and the underlying motivations: A comparison between household and hotel settings. Int. J. Hosp. Manag..

[104] Dolnicar S., Leisch F. (2008). An investigation of tourists’ patterns of obligation to protect the environment. J. Trav. Res..

[105] Dolnicar S., Leisch F. (2008). Selective marketing for environmentally sustainable tourism. Tour. Manag..

[106] Dolnicar S., Grün B. (2009). Environmentally friendly behavior: Can heterogeneity among individuals and contexts/environments be harvested for improved sustainable management?. Environ. Behav..

[107] Patronato Turismo Gran Canaria (2019). Situación del Sector Turístico año. https://www.grancanaria.com/turismo/es/.

[108] Sekaran U., Bougie R. (2016). Research Methods for Business: A Skill Building Approach.

[109] Gliem J.A., Gliem R.R. Calculating, interpreting, and reporting Cronbach’s alpha reliability coefficient for Likert-type scales. Proceedings of the Midwest Research-to-Practice Conference in Adult, Continuing, and Community Education.

[110] Aryal C. (2020). Exploring Circularity: A Review to Assess the Opportunities and Challenges to Close Loop in Nepali Tourism Industry. J. Tour. Adventure.

[111] Zhang Y., Tian L. (2014). The sustainable development of circular economy under the perspective of ecological tourism. Adv. Mater. Res..

